# Supplementary material for: An assessment for health education and health promotion in chronic disease demonstration districts: a comparative study from Hunan Province, China
Source: PeerJ. 2019 Mar 7;7:e6579. doi: 10.7717/peerj.6579 (PMC6409084; doi:10.7717/peerj.6579)
Supplement: Table S2 [file peerj-07-6579-s002.docx]

**Table S2. The original values of quality indicators in the ten sample districts (A–J)****

| **New seq.** | **Old seq.**  (“Raw 2”) | **A** | **B** | **C** | **D** | **E** | **F** | **G** | **H** | **I** | **J** |
| --- | --- | --- | --- | --- | --- | --- | --- | --- | --- | --- | --- |
| c1 * | 1 | 1 | 1 | 1 | 1 | 1 | 1 | 0 | 0 | 0 | 0 |
| c2 * | 2 | 1 | 1 | 1 | 1 | 1 | 1 | 0 | 0 | 0 | 0 |
| c3 * | 3 | 1 | 1 | 1 | 1 | 1 | 1 | 1 | 1 | 1 | 1 |
| c4 | 5 | 2436.5 | 943.1 | 149.8 | 730.1 | 319.5 | 1041.1 | 0 | 14.29 | 0 | 0 |
| c5 | 6 | 96 | 19.6 | 34.07 | 41.94 | 17.74 | 58.32 | 2 | 2 | 3 | 4.3 |
| c6 | 7 | 23.6 | 12.47 | 16.03 | 15.09 | 10.91 | 27 | 2.67 | 1.24 | 2.92 | 4.99 |
| c7 | 8 | 0.071 | 0.056 | 0.052 | 0.052 | 0.07 | 0.095 | 0.027 | 0.034 | 0.052 | 0.087 |
| c8 * | 10 | 1 | 1 | 1 | 1 | 1 | 1 | 0 | 0 | 0 | 0 |
| c9 * | 11 | 1 | 1 | 1 | 1 | 1 | 1 | 0 | 0 | 0 | 0 |
| c10.1 | 12 | 19 | 2 | 23 | 11 | 44 | 11 | 0 | 0 | 5 | 4 |
| c10.2 | 12 | 5 | 8 | 3 | 2 | 30 | 5 | 0 | 0 | 5 | 4 |
| c11 | 14 | 24 | 12 | 18 | 9 | 10 | 20 | 9 | 5 | 6 | 8 |
| c12 | 16 | 30 | 4 | 22 | 6 | 18 | 6 | 5 | 6 | 6 | 4 |
| c13 | 17 | 20 | 10 | 13 | 7 | 7 | 12 | 4 | 5 | 11 | 1 |
| c14 | 20 | 100 | 100 | 100 | 89.98 | 100 | 92.5 | 82.6 | 100 | 100 | 81 |
| c15 | 21 | 0.5 | 0.5 | 0.5 | 0.5 | 0.5 | 1 | 0.25 | 0.25 | 0.5 | 0.25 |
| c16 | 22 | 100 | 93 | 100 | 89.98 | 100 | 100 | 32.5 | 80 | 100 | 77 |
| c17 | 23 | 4.4 | 4.3 | 0.3 | 4 | 1.9 | 4 | 2 | 1 | 3 | 1 |
| c18 | 24 | 4 | 8 | 4 | 4 | 4 | 4 | 4 | 5 | 2 | 3 |
| c19 | 25 | 100 | 100 | 100 | 100 | 100 | 100 | 100 | 100 | 0 | 95 |
| c20 | 26 | 100 | 100 | 100 | 100 | 100 | 100 | 100 | 100 | 0 | 100 |
| c21 | 29 | 58.93 | 56.42 | 42.26 | 37.36 | 29.67 | 58.29 | 22.26 | 34.95 | 20.62 | 32.04 |
| c22 | 30 | 51.39 | 48.11 | 37.38 | 40.72 | 38.18 | 54.42 | 32.19 | 40.38 | 32.11 | 41.62 |
| c23 * | 35 | 1 | 1 | 1 | 1 | 1 | 1 | 0 | 1 | 0 | 0 |
| c24 | 36 | 86.26 | 73.86 | 81.96 | 80.85 | 71.83 | 87.16 | 78.40 | 76.35 | 80.67 | 80.76 |
| c25 | 38 | 10.86 | 10.42 | 9.79 | 9.29 | 8.45 | 7.96 | 6.92 | 9.55 | 1.25 | 2.50 |

* Qualitative items, of which positive items were defined as 1 and negative items defined as 0.

** The name of the districts (A–J) were detailed as: Furong District (A), Ziyang District (B), Shaodong County (C), Shuangfeng County (D), Luxi County (E), Yuhua District (F), Anhua County (G), Xinhua County (H), Xinshao County (I), and Jishou County (J).
