# Supplementary material for: An assessment for health education and health promotion in chronic disease demonstration districts: a comparative study from Hunan Province, China
Source: PeerJ. 2019 Mar 7;7:e6579. doi: 10.7717/peerj.6579 (PMC6409084; doi:10.7717/peerj.6579)
Supplement: Supplemental Information 2 [file peerj-07-6579-s004.docx]

致专家信

尊敬的专家：

您好！首先，非常感谢您百忙之中抽空赏顾我们的研究项目，在此也衷祝您工作如意、生活幸福安康！

我们的项目名称是慢性非传染性疾病（简称慢性病）综合防控示范区健康教育健康促进的效果评估及优化对策研究。下面，项目组就研究的主要内容，及与您可能相关的事项向您作一简短介绍。

2010年10月底卫生部在全国范围内启动慢性病综合防控示范区创建，我省各地也积极踊跃申报创建，但创建以来，如何有效评估示范区创建成果,成为丞待解决的一项重要工作。健康教育和健康促进为示范区创建的一项重要工作内容，其作为国际公认的低投入、高效益的慢性病防控战略，在发达国家起步较早，但在我国工作起步较晚，效果评价较少。基于此,本研究以示范区创建为契机，以健康教育和健康促进为切入点，采用定性与定量相结合的综合评价方法，构建效果评估指标体系，并评估其效果，为建立适应我省慢性病防控需求的健康教育与健康促进模式、提供科学依据和经验参考。

其中在构建评估指标体系这块，我们主要采用了德尔菲法（delphi法，又为专家咨询法），具体通过采取匿名信的方式广泛征求专家的意见,经过多轮咨询和反馈修正，使专家的意见趋于一致,最后根据专家的综合意见，对评价对象作出定性和定量相结合的预测与评价。

在应用德尔菲法前，我们在查询既往国内外类似项目研究，并在参鉴卫生部慢性病综合防控示范区工作手册等基础上，初步筛选了一些拟评指标，并制成专家咨询表（附后），请您就指标是否具有科学性、合理性、可操作性等，做出判断，并填写在表格中，必要时，请列出您的意见理由。并请您在填写表格完毕后，于二周内邮寄回：湖南省长沙市芙蓉中路1段450号，湖南省疾控中心慢性病防治科，徐巧华 收，邮编410005。
